# Feasibility and physiological relevance of designing highly potent aminopeptidase-sparing leukotriene A4 hydrolase inhibitors

**DOI:** 10.1038/s41598-017-13490-1

**Published:** 2017-10-19

**Authors:** Shin Numao, Franziska Hasler, Claire Laguerre, Honnappa Srinivas, Nathalie Wack, Petra Jäger, Andres Schmid, Arnaud Osmont, Patrik Röthlisberger, Jeremy Houguenade, Christian Bergsdorf, Janet Dawson, Nathalie Carte, Andreas Hofmann, Christian Markert, Liz Hardaker, Andreas Billich, Romain M. Wolf, Carlos A. Penno, Birgit Bollbuck, Wolfgang Miltz, Till A. Röhn

**Affiliations:** 1 Chemical Biology & Therapeutics, Novartis Institutes for BioMedical Research, Novartis Pharma AG, Basel, Switzerland; 2 Autoimmunity, Transplantation and Inflammation, Novartis Institutes for BioMedical Research, Novartis Pharma AG, Basel, Switzerland; 3 Analytical Sciences & Imaging, Novartis Institutes for BioMedical Research, Novartis Pharma AG, Basel, Switzerland; 4 Global Discovery Chemistry, Novartis Institutes for BioMedical Research, Novartis Pharma AG, Basel, Switzerland; 5 Respiratory Research, Novartis Institutes for BioMedical Research, Novartis Pharma AG, Basel, Switzerland

## Abstract

Leukotriene A4 Hydrolase (LTA4H) is a bifunctional zinc metalloenzyme that comprises both epoxide hydrolase and aminopeptidase activity, exerted by two overlapping catalytic sites. The epoxide hydrolase function of the enzyme catalyzes the biosynthesis of the pro-inflammatory lipid mediator leukotriene (LT) B4. Recent literature suggests that the aminopeptidase function of LTA4H is responsible for degradation of the tripeptide Pro-Gly-Pro (PGP) for which neutrophil chemotactic activity has been postulated. It has been speculated that the design of epoxide hydrolase selective LTA4H inhibitors that spare the aminopeptidase pocket may therefore lead to more efficacious anti-inflammatory drugs. In this study, we conducted a high throughput screen (HTS) for LTA4H inhibitors and attempted to rationally design compounds that would spare the PGP degrading function. While we were able to identify compounds with preference for the epoxide hydrolase function, absolute selectivity was not achievable for highly potent compounds. In order to assess the relevance of designing such aminopeptidase-sparing LTA4H inhibitors, we studied the role of PGP in inducing inflammation in different settings in wild type and LTA4H deficient (LTA4H KO) animals but could not confirm its chemotactic potential.  Attempting to design highly potent epoxide hydrolase selective LTA4H inhibitors, therefore seems to be neither feasible nor relevant.

## Introduction

LTA4H is a broadly expressed, cytosolic zinc (Zn) metalloenzyme that is part of the 5-lipoxygenase (5-LO) pathway which converts arachidonic acid (AA) into pro-inflammatory leukotrienes and anti-inflammatory lipoxins^1^. LTA4H catalyzes the final and rate limiting step in the biosynthesis of pro-inflammatory LTB4, that is, the vinylogous hydrolysis of the epoxide, LTA4. LTA4 binds to a deep L-shaped hydrophobic channel which forms one of two lobes of the catalytic site of the enzyme. In between the two lobes, the Zn^2+^ ion is located, complexed by the sequence HEXXH-(X)_18_E, a motif characteristic of M1 metallopeptidases^2^. LTA4H is well known for its epoxide hydrolase function because LTB4 is an important pro-inflammatory lipid mediator, capable of initiating and amplifying innate and adaptive immune responses. It acts as chemoattractant and stimulator of inflammatory mediator release by immune cells and has a particularly strong effect on recruitment and activation of neutrophils^3,4^. LTB4 has been implicated in many acute and chronic inflammatory diseases in humans and inhibition or knockout of LTA4H proved beneficial in numerous preclinical models of neutrophil-driven inflammation^5^. LTB4 is mainly produced by myeloid cells, in particular, neutrophils, macrophages, monocytes, and mast cells which also express the upstream enzyme 5-LO and 5-LO activating protein (FLAP) which convert AA into LTA4. LTA4H is additionally found in other cell types such as epithelial cells, endothelial cells, fibroblasts, keratinocytes, and erythrocytes and has been detected in almost all mammalian tissues and organs^6^.

The second lobe of the catalytic site of LTA4H is a more open and hydrophilic pocket, and forms the aminopeptidase activity of the enzyme. It hydrolyses tripeptides and has a high affinity for those with an N-terminal arginine^7^. While less is known about the physiological relevance of the aminopeptidase activity of LTA4H, Snelgrove and co-workers suggested that it can degrade the tripeptide PGP which has been reported to be a collagen-derived matrikine and neutrophil chemoattractant^8–10^. These data suggest that LTA4H may have two opposing roles in the regulation of inflammation: on the one hand, LTA4H is responsible for biosynthesis of the pro-inflammatory, neutrophil chemotactic lipid mediator LTB4 and on the other hand, it degrades and inactivates the chemotactic tripeptide PGP^9,11^. The authors of this study proposed that epoxide hydrolase specific LTA4H inhibitors sparing the aminopeptidase function may have superior anti-inflammatory activity because they would not interfere with the degradation of PGP while inhibiting LTB4 biosynthesis.

Since most reported LTA4H inhibitors extend from the hydrophobic pocket into the aminopeptidase binding site, they do not exhibit any epoxide hydrolase specificity^5^. Recent reports, however, have described small fragments that utilize almost exclusively the hydrophobic LTA4 binding site. These fragments were claimed not to interfere with PGP degradation or to even augment the peptidase activity of the enzyme^12–15^.

Encouraged by these reports, we initiated a drug discovery program with the aim to investigate the hypothesis that an aminopeptidase-sparing inhibitor may be superior to a compound inhibiting both activities. To identify highly potent and epoxide hydrolase-selective LTA4H inhibitors, we attempted to optimize the recently published compounds with reported selectivity in addition to conducting a high throughput screening (HTS) and structure-based rational drug design. This let us to the identification of compounds with some preference for the epoxide hydrolase pocket of the enzyme but no absolute selectivity versus the aminopeptidase pocket.

Furthermore, we studied the role of PGP in different inflammatory settings in wild type (wt) and LTA4H KO mice but were unable to confirm a chemotactic or pro-inflammatory role of PGP in neither mice nor human cells. Notably, published studies with LTA4H KO mice as well as reports with previously described aminopeptidase-interfering LTA4H inhibitors unanimously demonstrated a strong anti-inflammatory effect, particularly in models of neutrophil-driven sterile inflammation, such as colitis, arthritis and asthma^16^. Our results therefore question the physiological relevance of the PGP-degrading activity of LTA4H.

## Results

### Aminopeptidase-sparing potential of LTA4H inhibitors

In order to determine the relevance of PGP in disease settings, we searched for tool compounds which would allow for pharmacological testing of the hypothesis put forth by Snelgrove and co-workers^9,11^. To this end, we attempted to optimize two different compound classes 4-(4-benzylphenyl) thiazol-2-amine (ARM1) and 4-Methoxydiphenylmethane (4-MDM) that were reported to inhibit the epoxide hydrolase activity, but not aminopeptidase activity of the enzyme^13,14^, hoping to improve on potency without compromising selectivity. However, similar to what was reported recently by Low *et al*.^15^, these compounds turned out to inhibit both activities of the enzyme and were not epoxide hydrolase selective. Indeed, we found that close and even smaller analogues of ARM1 and 4-MDM also failed to show any selectivity for the epoxide hydrolase function of the enzyme (Table 1).

**Table 1:** Inhibition of surrogate substrates Arg-AMC, Pro-AMC, Ala-AMC or PGP hydrolysis by LTA4H inhibitors. Results are depicted as average IC_50_ value of a minimum of two independent biological replicates generated from 4 technical replicates at each concentration of test compound. The Ala-AMC column indicates whether compound increased or inhibited hydrolysis of Ala-AMC by the enzyme. The last column shows the ratio of the IC_50_ values of PGP versus Arg-AMC. Values >1 indicate selectivity towards the aminopeptidase activity of LTA4H.

Cpd-Nr.		IC_50_ Arg-AMC [μM]	IC_50_ Pro-AMC [μM]	Effect on Ala-AMC hydrolysis	IC_50_ PGP [μM]	Ratio IC_50_ PGP/Arg-AMC
4-MDM		10.12	33.635	increase	4.22	0.42
ARM-1		0.97	1.03	increase	0.39	0.40
1		0.092	0.116	increase	0.01	0.11
2		0.27	0.28	increase	0.38	1.4
3		6.89	5.89	increase	5.68	0.82
4		0.027	0.040	increase	0.01	0.37
5		13.00	5.43	increase	3.53	0.27
6		0.064	0.077	increase	0.045	0.70
7		2.87	1.69	increase	1.40	0.49
8		1.30	1.35	increase	1.87	1.44
9		0.48	0.47	increase	0.035	0.07
10		4.45	4.05	increase	1.97	0.44

### HTS to identify LTA4H inhibitors sparing aminopeptidase activity

**Figure 1:**
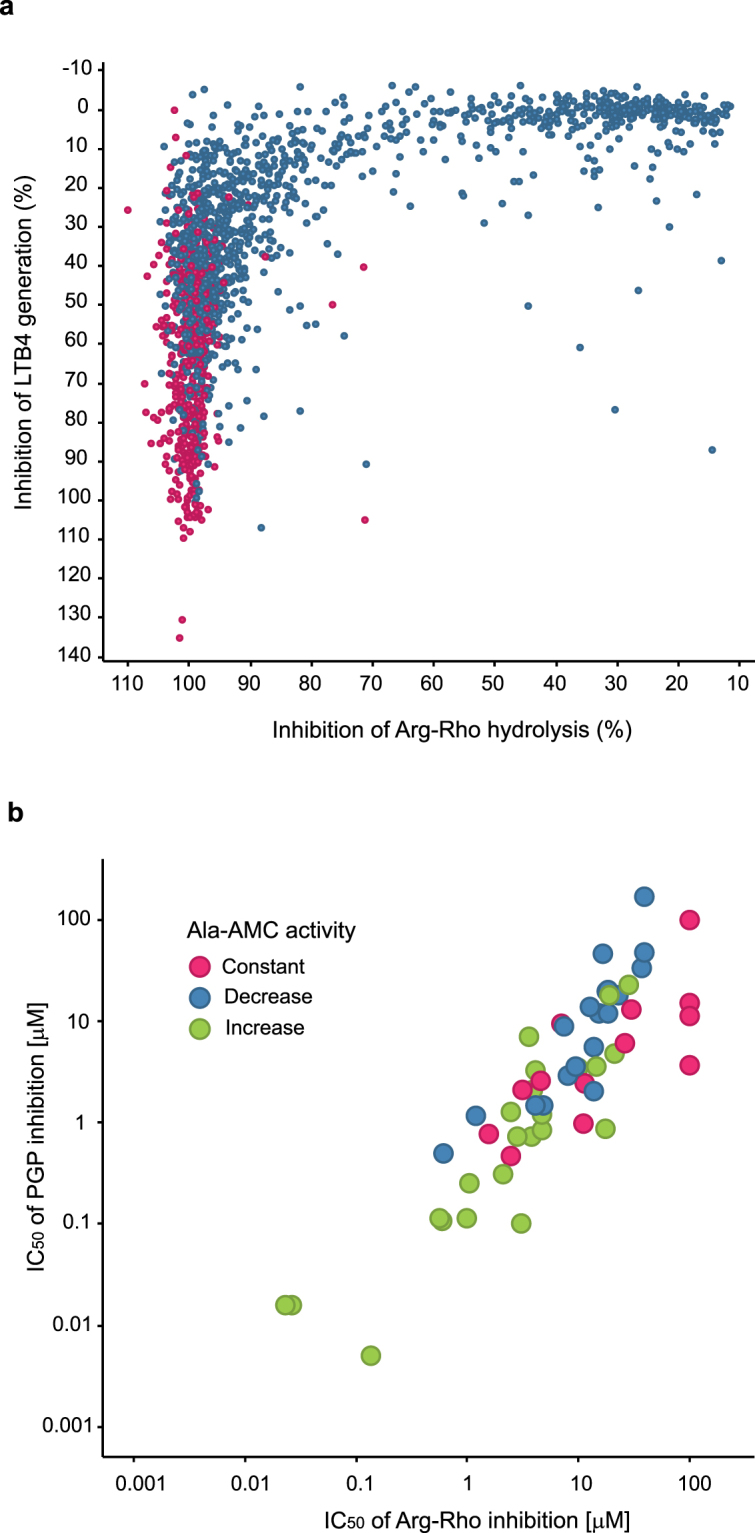
Potency of LTA4H HTS hits against various activities of LTA4H. The hits from the screen using LTA4 and Arg-Rho as substrates were re-tested in quadruplicates at 25 µM. **(a)** The results are depicted as average percent inhibition with all compounds that stabilizing the LTA4H protein in DSF (>3 standard deviation) colored in red. **(b)** Comparison of IC_50_ values of HTS hits sparing Pro-AMC or Ala-AMC activity using the Arg-Rho and PGP assays. The color indicates the effect of respective compounds in the Ala-AMC assay.

As the literature compounds and their derivatives did not selectively inhibit the epoxide hydrolase activity in our hands, we searched for new starting points by running an LTA4H inhibitor HTS. To this end, we performed a screen of 50.000 compounds using the surrogate substrate (see supplementary text for rational on substrates), Arginine Rhodamine (Arg-Rho) which probes a large portion of both active sites^17^. However, to avoid missing any compounds that bind exclusively to the deep portion of the hydrophobic pocket of the LTA4H active site, we also decided to run a parallel screen using an assay with LTA4 as the substrate. To confirm that hits identified in either the LTA4 screen or Arg-Rho screen inhibited LTA4H by binding to the enzyme, we tested all compounds by Differential Scanning Fluorimetry (DSF) to see whether they stabilized the protein. For all compounds active in either screen, the IC_50_ values were determined from dose response experiments using LTA4 and Arg-Rho as substrates. We identified over 300 compounds with IC_50_ values below 1 µM in the Arg-Rho assay and stabilizing the protein in the DSF assay. Comparison of the hit lists from the LTA4 and the Arg-Rho screens identified no additional hits from the LTA4 screen (Fig. 1a). The IC_50_ values of the hits were also determined using Proline-7-amino-4-methylcoumarin (Pro-AMC) and Ala-AMC as surrogate substrates for PGP hydrolysis, and of these, those that did not inhibit either assays were re-tested using PGP as the substrate. Comparing the IC_50_ values from the various assays, we found that, in spite of its extensive use as a PGP analogue in literature, the Ala-AMC assay did not predict whether the compounds spared PGP activity. Indeed, most tested compounds inhibited the PGP assay with a similar potency to that of the Arg-Rho assay (Fig. 1b).

### Rational design of inhibitors that spare the aminopeptidase pocket of LTA4H

As the unbiased approach did not yield a compound sparing the aminopeptidase activity, we decided to test whether we could obtain a potent aminopeptidase-sparing inhibitor by truncating an optimized LTA4H inhibitor, JNJ-26993135^18^, with the hypothesis that the potency against the epoxide hydrolase activity was not strictly coupled to potency against the aminopeptidase activity.

**Figure 2:**
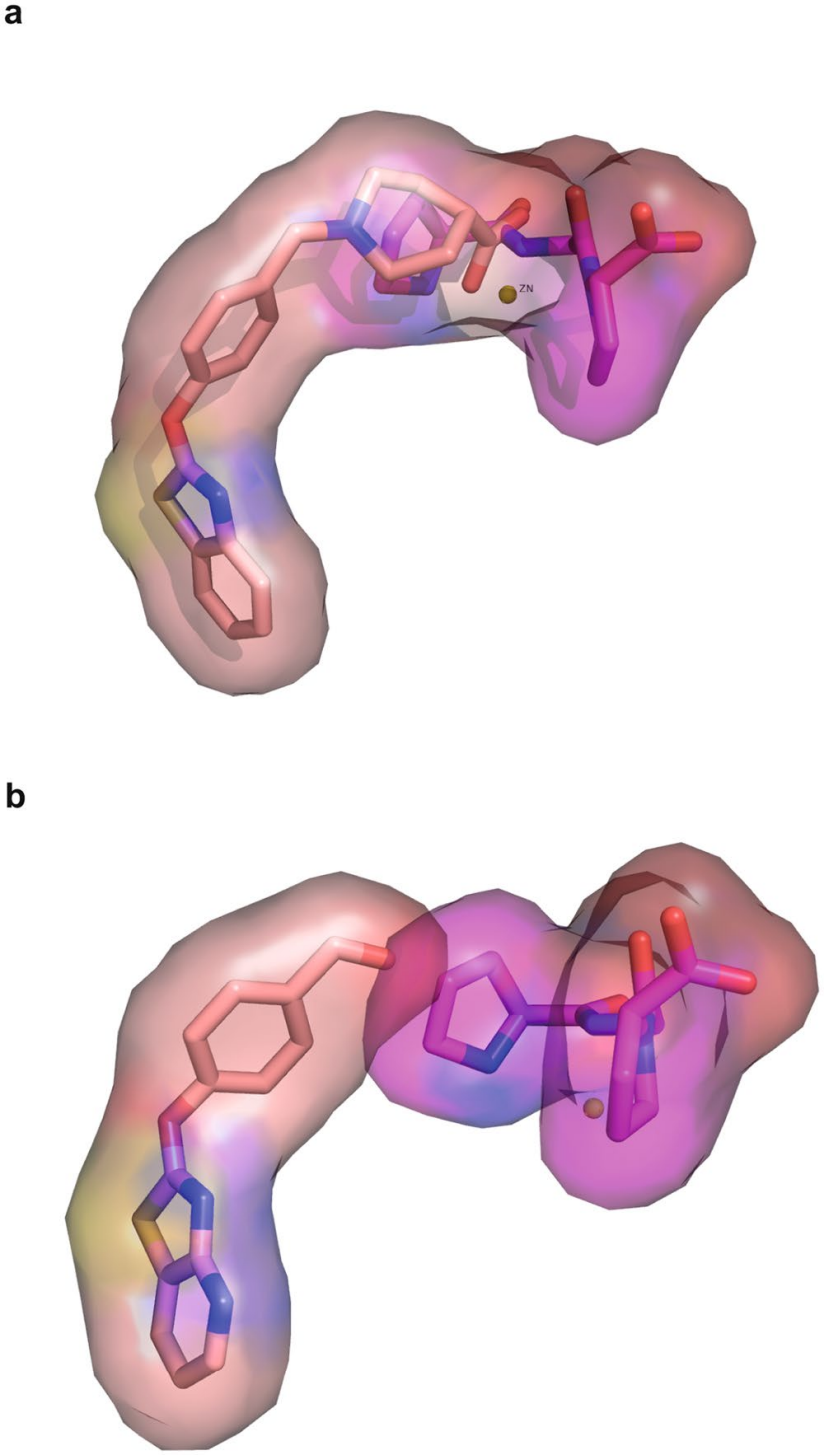
Position of compounds 11 and 15 within the active site of LTA4H. (**a)** Overlay of the x-ray structure of compound 11 in wt LTA4H with the structure of PGP in the inactive E296Q mutant LTA4H. Compound 11 extends far into the peptidase pocket of the enzyme and overlaps with the binding site of the tripeptide PGP. This steric interference leads to the potent inhibition of PGP hydrolysis. **(b)** Overlay of the x-ray structure of compound 15 in wt LTA4H with that of PGP in E296Q mutant LTA4H: the truncated compound 15 fully occupies the hydrophobic binding site of the enzyme but overlaps significantly less with the binding position of PGP, explaining the partial selectivity.

Overlaying the structure of LTA4H bound JNJ-26993135 (compound 11, Table 2) to that of PGP bound in the active site showed that simultaneous binding of the two ligands would not be possible due to steric effects (Fig. 2a). However, we hypothesized that by removing the part of the compound which interacts with the Zn^2+^, we would be able to have simultaneous binding of the PGP and the truncated compound to LTA4H. Removing the carboxylic acid group of JNJ-26993135 led to a compound that was still a potent epoxide hydrolase inhibitor (compound 12), but also did not spare the aminopeptidase activity. Replacement of the piperidine-4 carboxylic acid by only a carboxylic acid moiety, led to a compound inactive in both assays (compound 13). Modifying this to a hydroxymethyl group (compound 14) resulted in a moderate loss in potency towards epoxide hydrolase activity but interestingly, also a complete loss of activity against Ala-AMC (Table 2). However, also this modification did not result in a compound sparing PGP degradation. Converting the hydroxymethyl group to an aldehyde resulted in potent inhibitors (compound 15, 16) with selectivity against Ala-AMC and an approximately 4-fold selectivity (with regard to IC_50_ values) versus PGP. Best selectivity was achieved with compound 17 with an IC_50_ value of 0.082 µM in the Arg-AMC assay but only 1.11 µM in the PGP assay, resulting in a 14-fold selectivity. Interestingly, this compound did not enhance Ala-AMC hydrolysis by the enzyme but rather inhibited it, demonstrating again that Ala-AMC is not a predictive substrate. Analysis of the co-crystal structure of LTA4H with compound 15 did not reveal why the aldehyde containing compounds (compounds 15–17) may have been more selective towards epoxide hydrolysis than other small compounds located in the hydrophobic pocket (Fig. 2b). We observed in initial molecular dynamics (MD) modelling studies that most ligands binding to the hydrophobic pocket are less confined to a very rigid binding mode, suggesting that they are able to move within the pocket (see supplementary information on MD modelling with compound 17). In fact, in all of the co-crystal structures we obtained for these compounds, we noticed that the B-factor for the compounds were very high, suggesting high mobility of the compound.

**Table 2:** Inhibition of surrogate substrates Arg-AMC, Pro-AMC, Ala-AMC and PGP hydrolysis by LTA4H inhibitors. Results are depicted as average IC_50_ value [μM] of minimum two independent biological replicates of IC_50_ value determination being generated from 4 technical replicates at each concentration of test compound. The last column shows the ratio of the IC_50_ values of PGP versus Arg-Rho. Values > 1 indicate selectivity versus the aminopeptidase function of LTA4H.


Cpd-Nr.	X	Y	Z	R	IC_50_ Arg-AMC [μM]	IC_50_ Pro-AMC [μM]	IC_50_ Ala-AMC [μM]	IC_50_ PGP [μM]	Ratio IC_50_ PGP/Arg-AMC
11	CH	CH	CH		0.039	0.021	0.033	0.019	0.50
12	CH	CH	CH		0.030	0.027	0.041	0.007	0.3
13	CH	CH	CH		>25	>25	>25	13.6	n/a
14	CH	CH	CH		0.225	0.152	>25	0.076	0.34
15	N	CH	CH		0.033	0.047	>25	0.133	4.0
16	CH	N	N		2.577	15.72	>25	11.23	4.4
17	N	N	CH		0.082	0.56	0.55	1.11	13.5

While we identified compounds that had some epoxide-hydrolase selectivity over degradation of PGP, we were not able to identify inhibitors which completely spared the PGP hydrolysis activity. We therefore investigated the physiological relevance of PGP and the capacity of LTA4H to degrade it.

### LTA4H is the major PGP degrading enzyme *in vivo* in mice and in human blood

**Figure 3:**
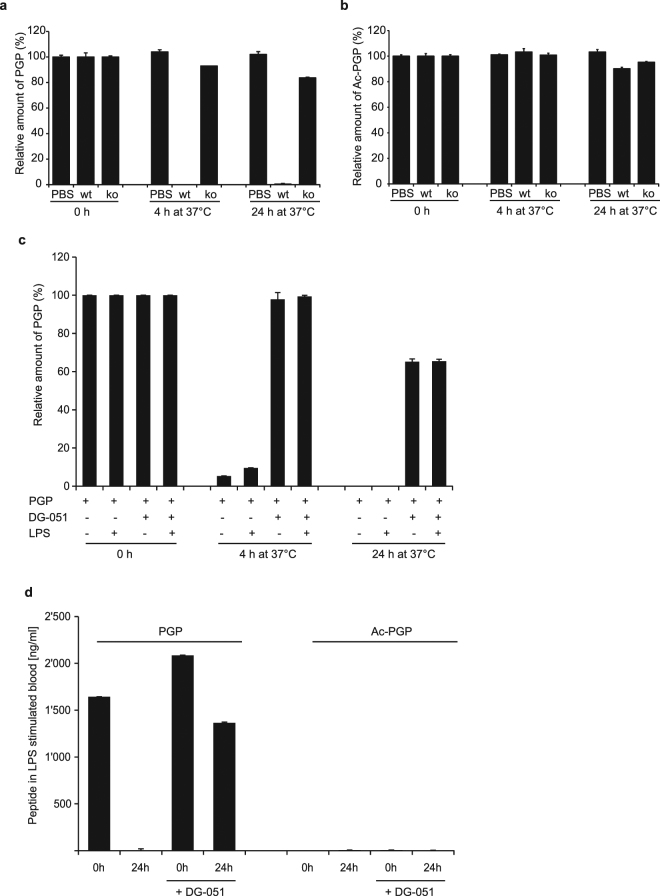
LTA4H is the dominant and non-redundant enzyme for PGP degradation in mouse and human. (**a)** PGP (10 nM) was spiked into PBS or BALF of wild type and LTA4H deficient mice and its degradation over time was monitored by LC-MS/MS. Values are given as percent of initial amount of PGP at time 0 h. Average ± SD from technical triplicates are given. **(b)** Ac-PGP was spiked into PBS or BALF of wild type and LTA4H KO mice and its degradation over time monitored by LC-MS/MS. Values are given as percent of initial amount of Ac-PGP at time 0 h. Depicted are averages ± SD from technical triplicates. **(c)** PGP was spiked into human blood, which was left untreated or stimulated with LPS and treated with or without the selective LTA4H inhibitor DG-051 (20 μM). PGP degradation over time was monitored by LC-MS/MS. Values are given as percent of initial amount of PGP at time 0 h. Depicted are averages ± SD from technical triplicates. **(d)** 2 μg/ml PGP were spiked into LPS stimulated human blood that was treated with DG-051 (20 μM) or left untreated as in (**c**). Depicted are the recovered amounts of PGP and Ac-PGP after 24 h of incubation in ng/ml as determined by LC-MS/MS. Values are given as averages of technical replicates ± SD.

To confirm that LTA4H is indeed the dominant and non-redundant enzyme capable of degrading PGP in mice and humans, we collected bronchoalveolar lavage fluid (BALF) from LPS challenged wt and LTA4H-KO mice^19^ and spiked them with PGP. In the BALF from wt animals, the added PGP was rapidly degraded and reached undetectable levels after only 4 h at 37 °C (Fig. 3a). On the other hand, in the exudate of the LTA4H KO mice, over 80% of the PGP was still present even after 24 h, suggesting that LTA4H is the dominant enzyme for degradation of PGP *in vivo*. Importantly, degradation of *N*-acetyl PGP (Ac-PGP), did not depend on LTA4H. When we spiked Ac-PGP into the exudates, no difference was observed between wt and LTA4H-KO mice (Fig. 3b). In both exudates, Ac-PGP remained as stable over 24 h at 37 °C as in PBS. This indicated that, as already observed by others^9^, LTA4H cannot degrade the acetylated version of PGP. We also monitored the potential formation of Ac-PGP in exudates after spiking PGP, but could not detect any Ac-PGP, indicating that the acetylated form is not generated in inflammatory exudates *in vitro* (data not shown).

To investigate whether the same would be true in humans, we spiked PGP into human blood and monitored its degradation over time. Human blood was either left untreated or stimulated with LPS. Some samples were treated with the potent and highly selective LTA4H inhibitor DG-051^20^. Degradation of PGP progressed rapidly and at the same rate in untreated and LPS stimulated blood, leading to complete disappearance of the tripeptide after 24 h at 37 °C (Fig. 3c). In the presence of DG-051 (20 µM), however, degradation was markedly slowed, leading to over 60% of initial PGP levels still remaining after 24 h. It has been published that human LPS stimulated neutrophils can acetylate PGP to generate Ac-PGP^21^. We therefore also monitored the levels of Ac-PGP in human blood stimulated with LPS and PGP, but could not observe any formation of Ac-PGP within 24 h under these conditions (Fig. 3d).

### PGP does not cause neutrophil influx in different *in vivo* models of acute sterile inflammation

**Figure 4:**
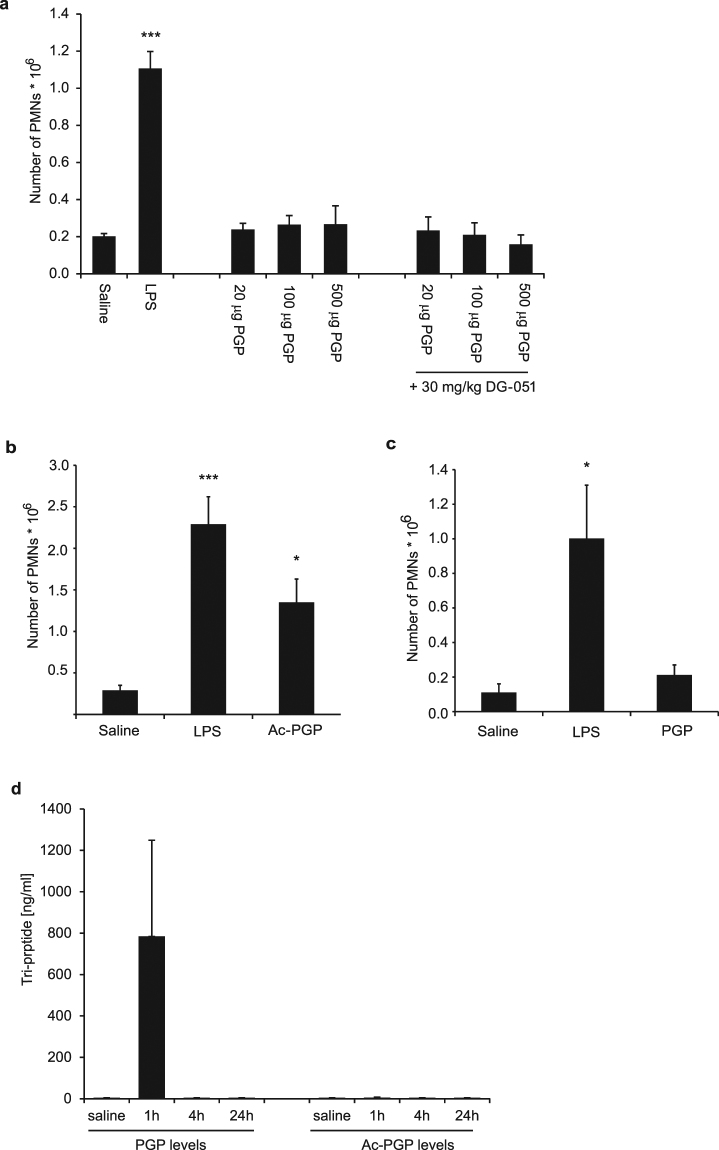
PGP does not induce neutrophil influx in murine air-pouch model. (**a**) Saline, saline containing LPS (10 μg/mouse) or increasing concentrations of PGP (20–500 μg/mouse) were applied to dorsal air-pouches of vehicle treated or DG-051 (30 mg/kg) treated female Balb/c mice. 4 h after induction, air-pouches were analyzed for neutrophil influx. Depicted are means (n = 6) ± SEM of one out of two experiments with comparable results. **(b)** Saline, saline containing LPS (10 μg/mouse) or Ac-PGP (250 μg/mouse) were injected into the dorsal air-pouches of female Balb/c mice. 4 h after induction, air-pouches were analyzed for neutrophil influx. Depicted are means (n = 8) ± SEM of one representative out of several experiments. **(c)** Saline, saline containing LPS (10 μg/mouse) or PGP (500 μg/mouse) were applied to dorsal air-pouches of LTA4H KO mice for 24 h. Depicted are means (n = 5) ± SEM. **(d)** PGP (500 μg/mouse) was injected into air-pouches of LTA4H KO mice and air-pouch exudate analyzed after 1 h, 4 h and 24 h by LC-MS/MS for content of PGP and Ac-PGP. Depicted are means (n = 5) ± SD. *P < 0.05, **P < 0.01, ***P < 0.001 Kruskal-Wallis followed by Dunn’s multiple comparison test post hoc.

Numerous literature reports investigating the role of LTA4H in sterile inflammatory conditions have demonstrated anti-inflammatory effects, using either LTA4H KO mice or pharmacological inhibitors. These effects were particularly pronounced in models of neutrophil-driven inflammation, such as arthritis, peritonitis, colitis and asthma^18,22–24^. Given the fact that LTA4H seems indeed to be the dominant enzyme that can degrade PGP *in vivo*, we wondered about the level of contribution PGP has to neutrophilic inflammation. We therefore studied the effect of PGP in different models of sterile inflammation. In a first model, we injected different concentrations of PGP into the dorsal air-pouch of mice and monitored cell influx. While LPS injection caused a robust inflammatory response and strong neutrophil influx, PGP up to a dose of 500 μg per air-pouch did not show elevated cell numbers compared to saline control (Fig. 4a). As the PGP injected into the air-pouch may have been degraded too quickly, we pre-treated mice with a high dose of 30 mg/kg of the potent LTA4H inhibitor DG-051 before injecting the tri-peptides into the air-pouches. Even with such a precaution, we were still unable to detect neutrophil influx (Fig. 4a). In contrast, when Ac-PGP was injected into the air-pouch, we observed a significant influx of neutrophils (Fig. 4b). We also injected 500 μg PGP into air-pouches of LTA4H KO mice to ensure complete abrogation of LTA4H activity, but again, we failed to detect significant cell influx in response to PGP (Fig. 4c). PGP concentrations in the air-pouches were monitored in these LTA4H deficient mice and while approximately 1 µg/ml of peptide could be determined in the exudate still 1 h after the injection, no PGP was detectable by 4 h and 24 h after the injection, possibly due to distribution into tissues and cells. Importantly, no Ac-PGP was detectable at any time after PGP injection, indicating that PGP does not seem to be acetylated *in vivo* either (Fig. 4d).

**Figure 5:**
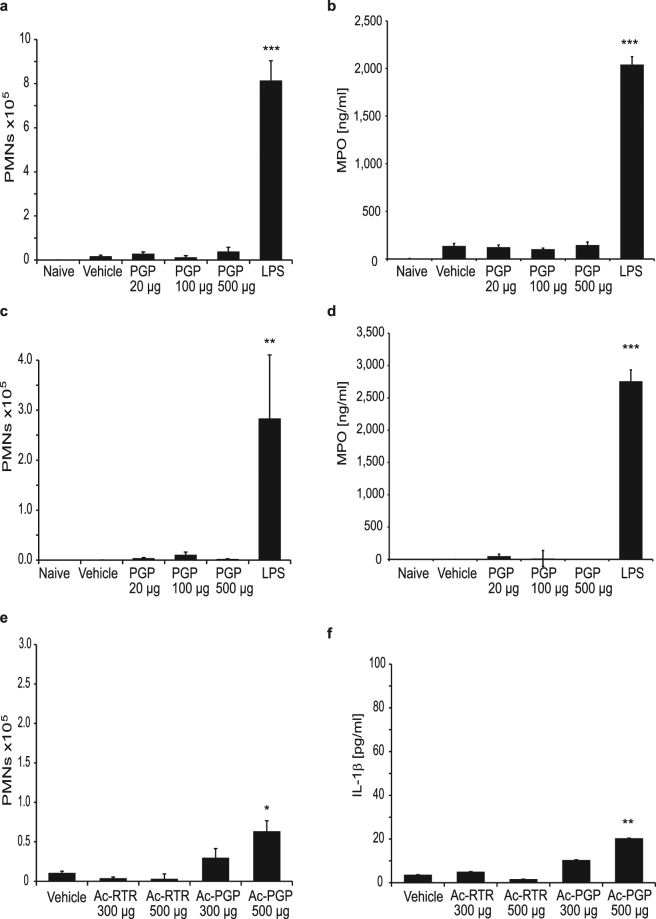
PGP does not induce neutrophil influx into murine lungs. Saline or saline containing LPS (10 μg/mouse) or increasing concentrations of PGP (20–500 μg/mouse) applied to lungs of female Balb/c mice by intra-tracheal administration. After 24 h, BALF was collected and cell influx analyzed by flow cytometry **(a)** and supernatants analyzed for MPO content by ELISA **(b)**. LTA4H KO mice were treated as described above and cell influx analyzed by flow cytometry **(c)** and supernatants analyzed for MPO content **(d)**. Female Balb/c mice were treated with saline or saline containing 300 and 500 μg/mouse of Ac-PGP or the control peptide Ac-RTR. Cell influx was analyzed by flow cytometry **(e)** or for IL-1β content by ELISA **(f)**. Data depicted as average (n = 6–10) ± SEM of one representative experiment each out of several performed in different mouse strains. *P < 0.05, ** P < 0.01, ***P < 0.001 Kruskal-Wallis followed by Dunns multiple comparison test.

Since the role of PGP as a pro-inflammatory mediator was mostly claimed in the context of pulmonary inflammation^9,11^, we extended our studies by injecting PGP into the lungs of wt or LTA4H KO mice. Different doses of PGP up to a dose of 500 μg were injected into the lungs of mice. Again, we were neither able to detect influx of neutrophils by flow cytometry of BALF cells nor elevation of myeloperoxidase (MPO) beyond the PBS control with any dose of PGP in either wt or LTA4H KO mice. By contrast, application of LPS led to strong increase in neutrophil numbers and MPO levels (Fig. 5a–d). When we injected Ac-PGP into the lungs of mice, we were able to detect a small, but significant influx of neutrophils at high doses of Ac-PGP in agreement with literature reports^25^. Also a significant elevation of inflammatory cytokines such as IL1-β was observed in response to Ac-PGP (Fig. 5e,f). These studies indicated that PGP, even in the absence of its main degrading enzyme LTA4H, does not show any pro-inflammatory potential *in vivo* in different settings. They also demonstrate that generation of significant levels of Ac-PGP, even from high concentrations of PGP, does not seem to occur *in vivo*.

### PGP is not chemotactic for human neutrophils

**Figure 6:**
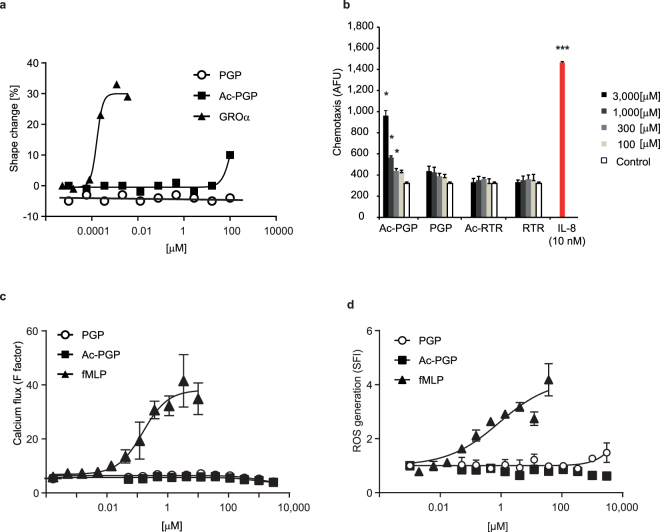
PGP is not chemotactic for human neutrophils and does not stimulate them. **(a)** Purified human neutrophils were incubated with increasing concentrations of PGP, Ac-PGP or GROα and induced shape change analyzed by flow cytometry. Results are depicted as dose response curves that are generated from 3 technical replicates at each concentration of test compound ± SD. **(b)** Chemotaxis of purified and calcein-AM labelled human neutrophils towards increasing concentrations of PGP, Ac-PGP as well as control peptides RTR and Ac-RTR as chemoattractants was determined after 90 min at 37 °C. Fluorescence in the receiving wells was measured. Depicted are means of fluorescence units (AFU) of 4 biological replicates ± SD. *P < 0.05 using the two-tailed Mann-Whitney test comparing PGP and AcPGP vs. same dose of respective control peptide. **(c)** Human neutrophils were stimulated with increasing concentrations of PGP, Ac-PGP or fMLP and induction of Ca^2+^ influx in response to stimuli was measured after cell labelling on a fluorescence reader. Results are depicted as dose response curves that are generated from 3 technical replicates at each concentration of test compound ± SD. Amplitude of calcium flux is given as specific fluorescence (F factor). **(d)** Human neutrophils were stimulated with increasing concentrations of PGP, Ac-PGP or fMLP. ROS generation was determined after labelling with dihydrorhodamine 123 by flow cytometry. Results are depicted as dose response curves that are generated from 3 technical replicates at each concentration of test compound ± SD. Amount of ROS generation is quantified as specific fluorescence index (SFI). All depicted experiments were reproduced with neutrophils from 4–6 human donors.

As we were not able to detect any chemotactic activity of PGP *in vivo* in different models of sterile inflammation, even in LTA4H KO mice, and only weak activity of Ac-PGP at very high doses, we went on to test the chemotactic activity of these two peptides on human neutrophils *in vitro*. Isolated human neutrophils were stimulated with ascending concentrations of PGP, Ac-PGP as well as the known chemokine GRO alpha (GROα) and neutrophil shape change was determined (Fig. 6a). While we observed a substantial shape change in response to GROα with an ED_50_ value of approximately 0.2 nM, no response to PGP could be observed up to 100 μM. At a concentration of 100 μM, Ac-PGP induced a very moderate response. We therefore tested both peptides in higher concentrations and compared them to two control peptides arginine-threonine-arginine (RTR) and its acetylated version (Ac-RTR), as well as the positive control, IL-8, in a neutrophil chemotaxis assay. As expected, IL-8 led to the induction of substantial chemotaxis at a concentration as low as 10 nM, while both control peptides were entirely negative (Fig. 6b). Ac-PGP triggered a mild but significant increase in chemotaxis above 300 μM which became more pronounced in the mM concentration ranges. By contrast, there was no significant increase in chemotaxis induced with PGP compared to the control peptide, RTR. We further extended our assessment of the physiological relevance of PGP and Ac-PGP on human neutrophils by testing for induction of Ca^2+^ flux or reactive oxygen species (ROS) which are both early and sensitive indicators for neutrophil activation. In contrast to the control tripeptide formyl-Met-Leu-Phe (fMLP), neither PGP nor Ac-PGP induced Ca^2+^ flux or ROS production in human neutrophil up to mM concentrations (Fig. 6c,d).

### PGP and Ac-PGP are present in sub-nanomolar concentrations in different models of lung inflammation

**Figure 7:**
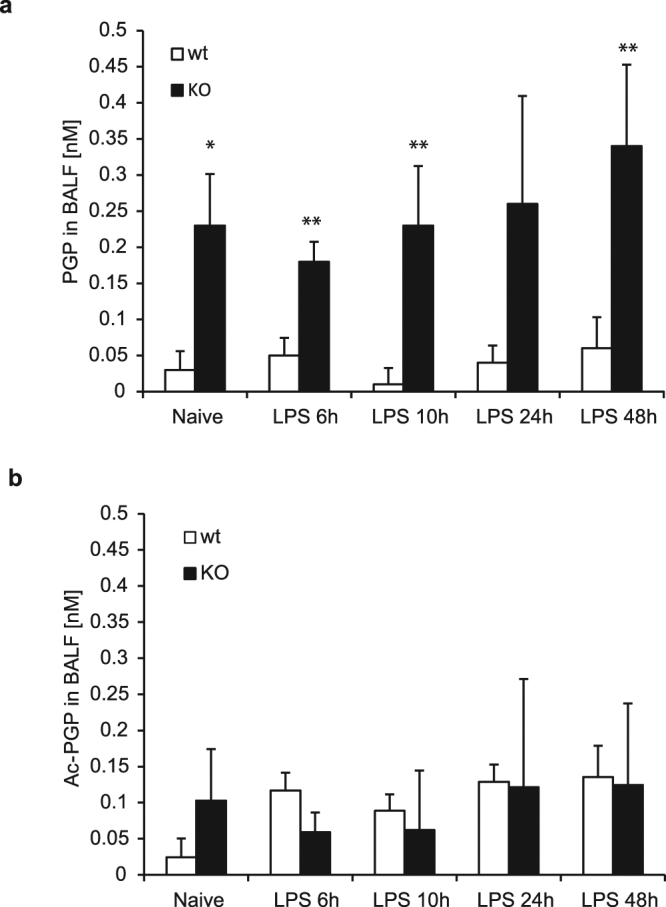
PGP but not Ac-PGP is elevated in BALF of LPS challenged lungs from LTA4H KO mice. Female wild type and LTA4H KO mice were stimulated via intra-tracheal administration of LPS (10 μg/mouse) and BALF collected after different time points to determine concentrations of PGP **(a)** and Ac-PGP **(b)** via LC-MS/MS. Depicted are means of 5 biological replicates ± SD. *P < 0.05, **P < 0.01 using the two-tailed Mann-Whitney test comparing wild type and LTA4H KO mice at each time point.

Given the inability of PGP to activate neutrophils *in vivo* and *in vitro* and up to mM concentrations we were interested in determining the levels of endogenous PGP in different inflammatory settings. Provided that the acetylated version of PGP showed some degree of pro-inflammatory activity at least in the high μM to mM range, we also determined the levels of Ac-PGP in these models. Others had determined nanomolar concentrations of PGP and Ac-PGP in murine BALF^9^ under inflammatory conditions and concentrations in the pM to nM range in human BALF^8^. We therefore quantified the level of PGP and Ac-PGP in BALF of wt and LTA4H deficient mice at different time points after intra-tracheal injection of LPS. While PGP levels were hardly detectable in BALF of wt mice, KO mice indeed showed an approximately 10 fold increase in PGP levels up to 0.3 nM in the observed time frame up to 48 h (Fig. 7a). Levels, however, remained sub-nanomolar even during these strongly inflammatory conditions. Concentrations of Ac-PGP were around 0.1 nM, close to the lower limit of detection (LLOD) and did not change with time. They were also not elevated in the LTA4H deficient mouse (Fig. 7b). In other models of airway inflammation, such as the ozone-induced inflammation or the tobacco smoke model, levels of PGP and Ac-PGP remained below the LLOD and could not be detected or quantified in our hands (data not shown).

## Discussion

Given the importance of LTB4 and its involvement in numerous chronic inflammatory as well as metabolic disorders, LTA4H is an attractive therapeutic target that has been pursued by numerous pharmaceutical companies in the past^26^. So far, however, no LTA4H inhibitor has reached the market. It has been speculated that LTA4H inhibitors that would spare the aminopeptidase activity of the enzyme would lead to superior and safer drugs^11,15^. We therefore undertook extensive studies to identify highly potent aminopeptidase sparing LTA4H inhibitors, including HTS and structure based drug design. While potent LTA4H inhibitors with some degree of epoxide hydrolase selectivity could be identified (compound 17), we could not identify potent inhibitors which completely spared the aminopeptidase activity. Somewhat surprisingly, although confirmed also by Low *et al*.^15^, was the finding that previously published epoxide hydrolase selective inhibitors were in fact unselective towards either activity. Our structural studies, as well as initial MD simulations suggested that many compounds are highly mobile within the hydrophobic binding pocket, adopting multiple binding modes. While we have no strong evidence, it is tempting to speculate that these multiple binding modes combine to give relatively small molecules such potency in the LTA4H active site. The corollary of this is that PGP may be able to simultaneously bind with compounds under certain conditions, albeit, in a potentially unproductive mode, such as in the case of ARM-1. A recent publication^15^ suggested that derivatives of reservatrol and isoflavone that interact with the deep end of the hydrophobic pocket as well as the Aspartate 375 residue showed an improved selectivity. It is conceivable that due to this anchoring, these compounds show less movement within the hydrophobic pocket and hence, interfere less with the enzymatic cleavage of PGP. However, possibly for the same reason, these compounds lack the potency seen with other LTA4H inhibitors.

As only limited selectivity can be gained for potent LTA4H inhibitors, we turned our interest towards investigating the physiological relevance of inhibiting the PGP degrading capacity of LTA4H. In contrast to literature suggestions^9,11,^ our results clearly demonstrate that the tripeptide PGP is not pro-inflammatory in different settings *in vivo* and also does not lead to the activation or chemotaxis of human neutrophils *in vitro* up to mM concentrations. Only its acetylated version Ac-PGP shows some activity at high concentrations. Importantly, these data are in agreement to earlier results obtained by Haddox *et al*. who showed that chemotaxis of human neutrophils can be induced with high concentrations of Ac-PGP with an EC_50_ value of approximately 0.5 mM while PGP is essentially inactive with an EC_50_ value of 15 mM^27^. Consistently, *in vivo* in both our air-pouch and lung inflammation models, we were unable to induce inflammatory responses even with very high concentrations of PGP. Even in mice treated with a high dose of an LTA4H inhibitor or in LTA4H KO mouse strains, PGP did not induce an inflammatory response, indicating that PGP as well as the ability of LTA4H to degrade it does not seem to have a physiological role in the mounting of an inflammatory response.

As Ac-PGP showed weak chemotactic potential, at least in the mM range, we wondered whether significant amounts of Ac-PGP might ever occur *in vivo* other than during the very special event of an alkali injured cornea model as in Pfister *et al*.^28^. Previous reports claimed that PGP may be converted to Ac-PGP by activated human neutrophils^21^. When we incubated LPS activated human blood with high concentrations of PGP, no Ac-PGP was formed over a period of 24 h, even when treating the blood with a LTA4H inhibitor which prevented PGP degradation. Similar observations were also made when spiking PGP into inflammatory exudates *ex-vivo* or when injecting large amounts of PGP into lungs or air-pouches of mice, indicating that under these conditions, PGP is not converted to Ac-PGP. Furthermore, we confirmed earlier observations^9^ that Ac-PGP cannot be degraded by LTA4H. Consequently, we did not observe an increase in Ac-PGP in the BALF of LTA4H KO mice after LPS induced inflammation. Importantly, careful determination of peptide concentrations revealed PGP as well as Ac-PGP were in the sub-nanomolar range even in the inflamed lungs of LTA4H KO mice and did not increase over time. In other models such as the ozone induced model of lung inflammation or the tobacco smoke model, no PGP or Ac-PGP could be detected, suggesting similar or lower concentrations of these peptides. Others have determined the concentrations of PGP or Ac-PGP in lungs of chronic obstructive pulmonary disease patients, cystic fibrosis patients or bronchiolitis obliterans syndrome patients^8,29,30^. Here again, the concentrations reported were in the low nM or even pM range, in agreement with our observations in the LPS induced lung inflammation model. These levels are significantly lower than the mM concentration of Ac-PGP required to induce neutrophil chemotaxis. Therefore, we have found that although LTA4H is the primary enzyme which degrades PGP, inhibition of LTA4H does not lead to the accumulation of PGP to a concentration where it may become chemotactic. Furthermore, under these conditions, PGP does not get converted to Ac-PGP. While our conclusions on the relevance of the PGP-degrading capacity of LTA4H are different from those of recent publications, we believe that they are not in conflict with the experimental results published initially on the biological activity of these peptides.

Recent reports demonstrated that LTA4H deficient mice show a stronger degree of neutrophilic lung inflammation after infection with influenza A  X31, Haemophilus influenzae b (Hib) or streptococcus pneumoniae than their wild type counterparts^9,11^. While it was speculated that this was due to elevated levels of PGP, even in these reports PGP was shown to be in the sub-nanomolar concentration range. In fact, only an association between increased neutrophil influx and an increase in PGP levels was shown. In addition, since the complementary peptide RTR also blocks the action of IL-8^25^, the reasoning that it reduces the neutrophilic influx does in our opinion not provide a proof for the causal effect of PGP in that study. Deficiency of leukotrienes in inflammatory lung conditions has previously been shown to impair proper host defense. Hence, the observed enhanced pulmonary inflammation and neutrophil influx could merely be explained by compensatory immune responses trying to overcome the deficiency in leukotrienes. This has for example been shown in models of fungal^31^ or Klebsiella pneumonia infections^32^ in 5-LO deficient mice, which - similar to LTA4H KO mice lack LTB4 - but clearly do not affect PGP degradation. Also in those pulmonary infection models, elevated neutrophil counts were detected in the 5-LO KO mice versus wild type counterparts. The elevated pulmonary inflammation in LTA4H KO mice may therefore also merely be a compensatory immune response to cope with the infection in the absence of LTB4. This would explain, why increase in neutrophilic inflammation in LTA4H deficient animals was only observed in models of pulmonary infection with pathogens. All other reports on sterile inflammatory conditions unanimously described a strong anti-inflammatory effect of LTA4H inhibition in models of neutrophil driven inflammation such as arthritis, colitis, peritonitis and asthma^18,22–24^.

Recent publications claimed that the compound 4-MDM would increase the aminopeptidase activity of LTA4H and hence lead to enhanced degradation of PGP^33^. Our results as well as those of Low *et al*., demonstrated that this compound actually inhibits PGP degradation in the same way as it inhibits LTB4 generation. The conclusions regarding the effects of this compound in lung emphysema models therefore cannot be explained by an elevated degradation of PGP and the conclusions with regards to the role of PGP as driver of the pathology in these studies are therefore ill-founded^33^. They may simply be explained by the role of LTB4 in lung emphysema as originally described^34^ and the observed effects seem to be due to partial inhibition of LTB4 biosynthesis with 4-MDM.

It has been speculated that LTA4H inhibitors may not have shown clinical efficacy due to the blocking of the PGP degrading capacity of the enzyme^11,15^. To our knowledge, only two LTA4H inhibitors have been tested in clinical efficacy trials to date. The first one, JNJ-40929837 by Johnson and Johnson, was tested in an asthma bronchial allergen challenge trial and did not show efficacy^35^. While there is little evidence for a role of LTB4 in asthma, the cysteinyl leukotrienes are important pathomechanistic drivers in allergen induced airway responses^36^. These were, however, elevated during treatment with JNJ-40929837, providing a potential explanation for the lack of efficacy. Linking this result to a potential PGP accumulation seems far-fetched and unsubstantiated. A second trial was done with the LTA4H inhibitor Acebilustat of Celtaxsys in cystic fibrosis patients^37^, a disease in which PGP levels were shown to be elevated^30^. In this trial, LTA4H inhibition reduced sputum neutrophils by 65%, despite the interference of this compound with PGP degradation. This questions the relevance of PGP and the capacity of LTA4H to degrade it even in a clinical setting. All other previously described LTA4H inhibitors by Decode, Johnson and Johnson, Searle, or Boehringer Ingelheim were stopped for reasons of compound-specific off-target toxicity, or discontinued for unknown reasons prior to clinical efficacy trials^26^.

LTB4 is a highly potent pro-inflammatory mediator and compounds that interfere with its biosynthesis have great promise in the treatment of inflammatory conditions. Our results demonstrate that the LTA4H substrate PGP is not chemotactic for human and mouse neutrophils. As originally described Ac-PGP shows moderate chemotactic effects but only at concentrations which can hardly be considered physiological. Ac-PGP cannot be degraded by LTA4H and it does not seem to be formed from PGP under inflammatory conditions. Although LTA4H is the dominant enzyme that can degrade PGP *in vivo*, the physiological relevance of this activity seems highly questionable. We conclude that there is no pharmacological benefit in LTA4H inhibitors that would spare PGP hydrolysis over those which are non-selective but potently inhibit both epoxide hydrolase and aminopeptidase activities.

## Materials and Methods

### Peptides

PGP (H-Pro-Gly-Pro) and Ac-PGP (N-acetly-Pro-Gly-Pro) were purchased from Bachem and Anaspec (AS-62009) as custom synthesis with a guarantee of less than 1 EU/mg endotoxins for *in vivo* experimentation. Fluorenylmethoxycarbonyl (Fmoc) -GP was also purchased from Bachem.

### Mice

8–12 week old wild type Balb/c and C57BL/6 mice (Charles River Germany Laboratories), as well as LTA4H KO mice on the 129/S6 (Jackson Laboratories) and BL/6 background (backcrossed at Novartis Pharma AG) were kept in specified pathogen free conditions in acclimatized animal rooms with 12 hours dark-light cycles. All animal studies were conducted according to the Swiss federal law for animal protection and animal protocols were approved by the authority of the Basel-Stadt Cantonal Veterinary Office, Switzerland.

### Human blood

Human blood was obtained from healthy volunteers in accordance with the guidelines of the Novartis Blood Donor Center, Basel. Informed consents of all blood donors were obtained and experimental protocols approved by the Novartis Blood Donor Center.

#### Enzyme expression and purification

Full-length LTA4H containing a N-terminal (His)_6_-tag followed by a (Gly_3_-Ser)_3_ spacer and a human rhinovirus 3 C (PreScission) cleavage site which leaves a Gly-Pro N-terminal overhang was expressed in E.coli BL21 (DE3) star cells. Cell lysates were applied to Nickle-NTA Superflow resin (Qiagen). The protein was eluted with a gradient of imidazole (20–500 mM) and was subjected to fusion tag removal by prescission site cleavage overnight. Finally, the LTA4H was further purified by size exclusion chromatography (Superdex SPX75/16/60; GE Health care). The resulting protein was estimated to be > 95% pure and homogeneous by SDS-PAGE and reverse phase HPLC. The identity of the protein was further confirmed by N-terminal sequencing and Mass spectrometry (Q-Tof, Micromass).

#### Co-crystallization of the LTA4H/ligand complex

Thin, plate-like crystals were obtained by the sitting drop vapor diffusion method at 17 °C after mixing 0.2 μL of LTA4H/ligand complex at 8 mg/ml protein concentration with an equal volume of reservoir solution consisting of imidazol (90 mM), yttrium chloride (5 mM), sodium acetate (74 mM), pH 6.5 in 96-well Cryschem plates. Crystals were harvested from their mother liquor with glycerol (20%) as cryoprotectant and directly flash-frozen in liquid nitrogen.

#### X-ray diffraction data collection and processing

Data sets were collected at 1.000 Å wavelength with a PILATUS 6 M detector at the Swiss Light Source beamline × 10SA (Villigen, Switzerland). Diffraction images recorded at 0.25° oscillation angle wedges were processed and scaled using XDS and XSCALE respectively, in the APRV program suite^38^. Data collection and processing statistics are summarized in supplementary Table 1.

#### Structure determination

An initial structure was obtained by molecular replacement using Phaser^39^ and the previously published coordinates of the protein component of the LTA4H structure (PDB entry 3FH5) as a search model. Manual rebuilding of the model and subsequent structure refinement were carried out in Coot^40^ and autoBuster^41^, respectively. The ligand structure was built into unbiased Fo-Fc difference electron density calculated by autoBuster. Final structure refinement statistics are summarized in supplementary Table 1. Refined coordinates were deposited in PDB (codes: 6ENB, 6ENC and 6END).

#### LTA4H enzyme assays

The LTA4H hydrolase assay was modified from the assay developed by Liang *et al*.^42^ to be compatible with 1536-well plates. Briefly, assays were performed at room temperature in Dulbecco’s phosphate buffer containing casein (0.1%), BSA (0.5%) and potassium fluoride (0.8 M). Compounds were incubated with LTA4H (10 nM) for 15 min prior to the addition of freshly deprotected LTA4 (deprotection of the methyl ester of LTA4 was performed in 80% DMSO −20% NaOH (0.25 M) (v/v) and then diluted 10 fold in 95% DMSO-5% NaOH (0.25 M) for a final concentration of 500 nM LTA4 in the well. After 30 minutes incubation, the reaction was stopped and the LTB4 levels measured by addition of the HTRF reagents (CisBio, France), LTB4-XL665 conjugate (1/100) and anti-LTB4-crytate conjugate (1/100) in DPBS buffer containing Casein (0.1%) and KF (0.8 M). The plates were incubated 2 h prior to measurement of the time-resolved fluorescence energy transfer (TR-FRET) signal on an EnVision reader. For the HTS, compounds were tested at 25 µM whereas for dose response experiments, the compounds were tested in a concentration range between 0.8 nM and 25 µM.

The LTA4H aminopeptidase assays were run using several surrogate substrates: Arg-Rho110-D-Pro (Arg-Rho, Biosyntan GmbH, Germany) or Arg-AMC (Bachem, Switzerland), Pro-AMC (Bachem, Switzerland) and Ala-AMC (Bachem, Switzerland). Assays were performed at RT in 1536-well plates in Tris buffer, pH 7.2 containing NaCl (100 mM), CaCl_2_ (2 mM), CHAPS (0.5%) and BSA (0.1%). Compound was incubated with LTA4H (20 nM) for 30 minutes prior to the addition of substrate (25 µM, 200 µM, 100 µM for Arg-Rho110-D-Pro, Pro-AMC, Ala-AMC, respectively). The reaction was incubated between 1–2 h (depending on substrate) prior to reading the increase in fluorescence on an EnVision. For the HTS, compounds were tested at 25 µM whereas for dose response experiments, the compounds were tested in a concentration range between 0.8 nM and 25000 nM.

#### PGP peptidolysis assay by LC-MS/MS

The aminopeptidase activity of LTA4H was assessed with H-PGP-OH (Bachem, Switzerland) as substrate. Assays were performed at RT in black 384-well low volume plates (Greiner) and analyzed by LC-MS/MS. All compounds were tested as 8 point dose response curves in triplicates included on every plate. 3 µl of compound diluted in assay buffer (Tris (50 mM), NaCl (150 mM), CaCl_2_ (10 mM) at pH7.5) was transferred to the plate followed by 3 µl of enzyme solution (20 nM LTA4H in assay buffer). The enzyme compound mixture was incubated at room temperature for 15 min prior to the addition of 6 µl substrate solution (800 µM PGP in assay buffer). Upon addition of the substrate, the plate was placed on a shaker at 25 °C for 40 min. The reaction was stopped by addition of 12 µl formic acid (0.2% v/v). The final concentration of the enzyme in the assay was 5 nM, the PGP 400 µM and the compound concentration range was from 50000 nM to 3.2 nM. For peptide quantification, an HPLC instrument from Agilent Series 1290 (binary high pressure gradient system) with detectors UV, fluorescence and mass (single Quad) detectors was used. HPLC was done using an Atlantis T3 column (C18 100 × 2.1mm, 3 µM, 100 Å) with A: water (MQ) + 0.1% formic acid and B: acetonitrile + 0.1% formic acid: 0–0.3 min 0% buffer B, then increased to 100% buffer B at 0.31–0.65 min and again decreased to 0% buffer B at 0.66–0.70 min. Flow was set to 1.5 ml/min with start gradient pressure of 400 bar. Mass transitions were at 116 for proline, 173 for glycine-proline and 270 for PGP.

#### PGP degradation assay in BALF *ex-vivo* and LC-MS/MS analysis

200 µl of BALF were spiked with PGP and Ac-PGP at the final concentration of 10 nM. Samples were then incubated at room temperature (RT) for 0 h, 4 h and 24 h. After incubation, samples were filtered (Amicon Ultra-0.5, Membrane Ultracel-30, PMNL 30 kD) by centrifugation (3 min at 10,000 × g). Filtered samples were transferred to vials for liquid chromatography tandem mass spectrometry (LC-MS/MS) analysis. The LC system consisted of an Acquity UPLC I-Class interfaced with a Xevo-TQS triple quadruple MS (Waters). Chromatographic separation of PGP and Ac-PGP was obtained using reverse-phase column (ACQUITY UPLC BEH130 C18 (2.1 × 50 mm, 1.7 µM) heated at 40 °C. The flow rate was set to 0.100 mL/min and the mobile phase consisted of water-formic acid (99.9/0.1; v/v) (A) and acetonitrile-formic acid (99.9/0.1; v/v) (B). The eluent gradient was set at 1% of B from 0–0.5 min, from 1%-99% of B during 0.5–3 min, and hold at 99% of B until 3.5 min. From 3.5–5 min the column was re-equilibrated with initial conditions. Retention times were typically 1.89 and 2.13 min for PGP and Ac-PGP respectively. Fragmentations were tuned for each compound individually in positive ion mode. PGP was monitored with MRM m/z transitions of 270.1 > 173.1 (quantifier) and 270.1 > 70.1 and 270.1 > 116.1 (qualifiers). Ac-PGP was monitored with m/z transitions of 312.2 > 112.1 (quantifier) and 312.2 > 116.1 and 312.2 > 140.1 (qualifiers).

#### PGP degradation assay in human blood

Fresh blood was collected in heparinized tubes by venipuncture from volunteers from the Novartis Blood Donor Center, Basel. The blood was diluted 1:3 in Roswell Park Memorial Institute (RPMI) containing HEPES (0.025 M). DG-051 was added to a final concentration of 20 µM and the plate was incubated for 15 min at 37 °C, 5% CO_2_ and 90% humidity. PGP and LPS were added at final concentrations of 2 µg/ml each and incubated up to 24 h at 37 °C, 5% CO_2_ and 90% humidity before samples were collected for LC-MS/MS analysis.

#### Human neutrophil shape change assay

Neutrophils were purified by Ficoll density gradient centrifugation from human blood and incubated with graded concentrations of PGP, Ac-PGP or the known neutrophil chemoattractant GROα in assay buffer containing BSA (0.1%) in PBS for 10 min at 37 °C, 5% CO_2_ and 90% humidity. Cells were then fixed and shape change analyzed by flow cytometry.

#### Human neurophil chemotaxis assay

Purified human neutrophils were labeled with calcein-AM (Fluka, Buchs, Switzerland). Test compounds (PGP, Ac-PGP, RTR, and Ac-RTR) or IL-8 (Sigma) as positive chemoattractant control were diluted in RPMI containing BSA (0.1%) and added at graded concentrations into the wells of a 96-well ChemoTX plate (3.2 mm diameter, 5 µm pore size; NeuroProbe, Gaithersburg, MD). Labeled cells (50,000 per well) were added onto the filters above the wells that received the test compounds. Plates were incubated for 90 min at 37 °C. The filters were removed and fluorescence in the receiving wells was measured using a SpectraMAX Gemini EM instrument (Molecular Devices, Sunnyvale, CA; λ_ex_ = 485 nm; λ_em_ = 538 nm). The chemotactic index was measured as the ratio of fluorescence in the test wells and in control (buffer only) wells.

#### Determination of ROS generation in human neutrophils

Purified human neutrophils were plated in RPMI + FCS (5%) in 96-well cell culture plates (100,000 cells/well). Dihydrorhodamine 123 (SIGMA) was added at a final concentration of 3 µM together with PGP, Ac-PGP and fMLP (SIGMA) at graded concentrations and incubated for 30 min at 37 °C, 5% CO_2_ and 90% humidity. Samples were then immediately put on ice and ROS generation quantified on a flow cytometer (BD LSR Fortessa) at excitation/emission: 488/525 nm. Amount of generated ROS is given as GMFI (sample)/GMFI (unstimulated control).

#### Human neutrophil calcium flux assay

Purified human neutrophils were incubated in Calcium 5 loading buffer (Molecular Devices) containing Probenecid (2.5 mM, SIGMA) at 37 °C, 5% CO_2_ and 90% humidity for 1 h. 20 µl of the cell suspension were then transferred to a black 384-well plate with clear bottom and centrifuged at 100 g for 4 min. Test compounds PGP, Ac-PGP and fMLP (SIGMA) were prepared at graded concentrations in HBSS containing HEPES (20 mM). Fluorescence was measured in FLIPR Tetra reader (Molecular Devices) every second before and after the addition of 5 µl of compounds dilutions. The calculated F index is the difference between the maximum signal and the average baseline signal, divided by the average baseline signal.

#### Air-pouch model of acute inflammation

Air-pouches were formed by subcutaneous injection of 5 ml air through a 0.22 μm filter on the backs of female LTA4H KO or wt mice. The patency of the cavity was maintained by a further 3 ml injection on day 3. Six days after the initial injection of air, LPS at 10 µg/animal and PGP, Ac-PGP, at different concentrations up to 500 µg/animal were injected into the air pouches. 4 h after the cell injections the mice were killed, 2 ml of sterile saline (containing 10 IU of heparin per ml) was injected into the intact pouch and the pouch gently massaged. An incision was made on the top of the pouch and the injected lavage fluid pipetted out into an eppendorf tube. An aliquot of air pouch lavage fluid was used to determine total cell counts on an Advia 120® Haematology Analyser. To determine differential cell counts, cytospins were prepared for all pouch fluid samples. The cytospins were air dried prior to staining with DiffQwik differential staining solutions. Differential cell counts were performed manually (x200 magnification) to determine the percentage of neutrophil and non-neutrophil cell populations in each sample.

#### Stimulation of lungs with PGP and Ac-PGP

PGP or Ac-PGP in increasing doses up to 500 ug per mouse were applied by intra-tracheal administration in 50 µl PBS to female LTA4H KO or wild type mice. After 24 h, the mice were euthanized and BALF was collected by cannulating the trachea with a catheter and lavaging the lungs 3 times with 0.4 ml cold PBS. The lavage fluid was then centrifuged and the cells analyzed by flow cytometry. Cell supernatants were used for analysis of MPO and cytokine content in BALF.

#### LPS induced lung inflammation model

LPS (10 ug per mouse) was applied by intra tracheal administration in 50 µl PBS to female LTA4H KO or wild type mice. At different time points after stimulation, mice were euthanized and BALF collected for analysis of PGP and Ac-PGP content in the BALF as described above.

#### Tobacco smoke exposure model

C57/Bl6 mice were exposed to either air (sham animals) or whole body smoke exposure (1R3F University of Kentucky research cigarettes) for 30 minutes. Briefly, smoke exposed animals received 4 seconds of smoke every 64 seconds of air at a flow rate of 0.6 l/min. In the afternoon, at least 5 h following the morning smoke exposure, animals were again exposed to either air or whole body smoke exposure for 30 minutes. The procedure was repeated for 6 weeks. 18 h after the last smoke exposure animals were euthanized and BALF collected for analysis of PGP or Ac-PGP as described above.

#### Analysis of endogenous PGP and Ac-PGP levels in BALF and inflammatory exudates by LC-MS/MS

100 µl of BALF were quenched in 11 µL of methanol-formic acid (99/1; v/v) containing Fmoc-GP used as a surrogate internal standard (ISTD) at the final concentration of 5 nM. After that, samples were vigorously mixed and centrifuged for 15 min at 16000 × g at RT. 100 µL of supernatants were transferred to vials and 5 µL of volume was injected. The LC-MS/MS system consisted of a Shimadzu Nexera × 2 interfaced with Sciex 65000 QTrap MS (Sciex). Chromatographic separation of PGP and Ac-PGP was obtained using reverse-phase column (Phenomenex Gemini C18 110 Å 2.0 × 50 mm, 5 µM) heated at 60 °C. The flow rate was set to 0.400 mL/min and the mobile phase consisted of water-formic acid (99.9/0.1; v/v) (A) and acetonitrile-formic acid (99.9/0.1; v/v) (B). The eluent gradient was set from 3%–98% of B during 0–3.5 min, and hold at 98% of B until 4.4 min. From 4.4–5.8 min the column was re-equilibrated with initial conditions. Retention times were typically at 0.55, 2.10 and 3.15 min for PGP, Ac-PGP and Fmoc-GP, respectively. All metabolites were analyzed in MRM mode with the following m/z transitions: PGP, 270.0 > 173.0, 270.0 > 69.9, 270.0 > 116.0, 270.0 > 155.1; Ac-PGP, 312.0 > 112.0, 312.0 > 116.0, 312.0 > 140.0, 312.0 > 69.9; Fmoc-GP, 395.0 > 173.1 and 395.0 > 179.1. The sum of transitions were quantified against a calibration curve (0.05 nM–200 nM) constructed in PBS.

#### Statistical Analysis

Statistical analysis were performed using the non-parametric Kruskal-Wallis test followed by Dunn’s test for multiple comparisons post hoc, and the Mann-Whitney test for comparison of two sample means. P values < 0.05 were considered significant. Sample numbers and experimental repetitions are indicated in respective figure legends.

### Compounds

The following compounds are commercially available: 4-MDM (CAS Nr. 8234–14–0, Matrix Scientific Cat Nr 3140), ARM-1 (CAS Nr. 68729–05–5, Matrix Scientific Cat Nr 135662), **1** (CAS Nr. 105512-82-1, Matrix Scientific Cat Nr 8070), **3** (Small Molecules Inc., Cat Nr. 62-1435), **4** (CAS Nr. 111273-31-5, Heterocyclics, Inc. Cat Nr. JR-3220), **10** (CAS Nr. 2215-78-3, ABCR GmbH, Cat Nr. AB354155), **11** (CAS Nr. 841202-16-2, $C Pharma Scientific Inc., Cat Nr. 4CH-031875), **14** (AbamaChem, Cat Nr. ABA-6720718). Compounds **5** ^43^, **12** ^44^, **13** ^45^ and **15** ^46^ are described in the literature. ^1^H NMR spectra were recorded on a Bruker 400 MHz NMR spectrometer. Chemical shifts are reported in parts per million (ppm) relative to an internal solvent reference. Significant peaks are tabulated in the order of multiplicity (s, singlet; d, doublet; t, triplet; q, quartet; quintet; m, multiplet; br, broad), coupling constants, and number of protons. Final compounds were purified to ≥ 95% purity as assessed by analytical liquid chromatography with the following method: Waters Acquity UPLC-MS; column HSS T3 1.8 μm, 2.1 × 50 mm; A, water + 0.05% formic acid + 3.75 mM ammonium acetate; B, acetonitrile + 0.04% formic acid; 5–98% B in 1.4 min, 98% B 0.45 min, flow 1.0 mL/min; column temperature 60 °C.

#### Synthesis of compound 2

**Step1:** 4-bromo-1-((2-(trimethylsilyl)ethoxy)methyl)-1H-imidazole. NaH (8.16 g, 60% in mineral oil, 204 mmol) was added at 0 °C to a solution of 4-bromo-1H-imidazole (10 g, 68.0 mmol) in 200 ml of DMF in three portions over 5 min and stirred for 30 min. Then (2-(chloromethoxy)ethyl)trimethylsilane (18.10 ml, 102 mmol) was added and the mixture was allowed to stir over night at rt. The reaction mixture was quenched with water and extracted three times with ethyl acetate. The combined organic layers were washed with brine, dried over Na_2_SO_4_, filtered and concentrated. The crude was purified by MPLC chromatography (silica gel, ethyl acetate/hexane 0–40%, 30 min). Yield: 11.6 g (61%) of a brown oil. ^1^H NMR (400 MHz, DMSO-*d*_6_): δ 8.03 and 7.84 (s, 1 H), 7.47 and 7.07 (s, 1 H), 5.34 and 5.36 (s, 2 H), 3.52 and 3.54 (t, J = 12 Hz, 2 H), 0.88 (t, J = 12 Hz, 2 H), 0.0 (s, 9 H); MS (ESI): 277.0, 279.0 [M + H]^+^; HPLC: t_R_ = 1.10 (32%) and 1.11 min (68%)(mixture of regioisomers).

**Step 2:** 4-(4-benzylphenyl)-1-((2-(trimethylsilyl)ethoxy)methyl)-1H-imidazole. To a solution of 4-bromo-1-((2-(trimethylsilyl)ethoxy)methyl)-1H-imidazole (150 mg, 0.541 mmol) and (4-benzylphenyl)boronic acid (229 mg, 1.082 mmol) in 10 ml of 1-propanol was added a 2 M aqueous Na_2_CO_3_ solution (1.35 ml, 2.71 mmol). The reaction mixture was flushed with argon and PdCl_2_(PPh_3_)_2_ (19 mg, 0.027 mmol) was added. The mixture was stirred for 16 h at 90 °C. The reaction mixture was partioned between ethyl; acetate and saturated NaHCO_3_ solution. The aqueous layer was extracted twice with ethyl acetate. The combined organic layers were washed with brine, dried over Na_2_SO_4_ and concentrated. The residue was purified by MPLC chromatography (silica gel, methanol/dichloromethane 0–60%, 13 min) to give 57 mg (89%) of product. MS (ESI): 365.2 [M + H]^+^; HPLC: t_R_ = 1.3 (56%) and 1.35 min (29%) (mixture of regioisomers).

**Step 3**: 4-(4-benzylphenyl)-1H-imidazole (**2**). TFA (98% in H_2_O, 2.5 mL, 32.5 mmol) was added dropwise over 5 min to 4-(4-benzylphenyl)-1-((2-(trimethylsilyl)ethoxy)methyl)-1H-imidazole (158 mg, 0.260 mmol) and stirred for 1.5 h at rt. The rxn mixture was treated with saturated NaHCO_3_ solution, diluted with dichloromethane and washed with water. The aqueous layer was extracted three times with dichloromethane. The combined organic layers were dried over Na_2_SO_4_, filtered and concentrated. The crude product purified by MPLC chromatography (silica gel, methanol/ethyl acetate 0–50%, 15 min) to give 148 mg (56%) of product. ^1^H NMR (400 MHz, DMSO-*d*_6_): δ 7.4–7.6 (m, *6 *H), 7.1–7.25 (m, 6 H), 3.85 (s, 2 H); MS (ESI): 235.1 [M + H]^+^; HPLC: t_R_ = 0.75 min (93%).

#### Synthesis of compound 6

5-(4-phenoxyphenyl)-1H-imidazole. To a solution of 5-bromo-1H-imidazole (50 mg, 0.340 mmol) and (4-phenoxyphenyl)boronic acid (146 mg, 0.680 mmol) in 10 ml of 1-propanol was added a 2 M aqueous Na_2_CO_3_ solution (0.85 ml, 1.7 mmol). The reaction mixture was flushed with argon and PdCl_2_(PPh_3_)_2_ (11.9 mg, 0.017 mmol) was added. The mixture was stirred for 16 h at 90 °C. The reaction mixture was partioned between ethyl; acetate and saturated NaHCO_3_ solution. The aqueous layer was extracted twice with ethyl acetate. The combined organic layers were washed with brine, dried over Na_2_SO_4_ and concentrated. The residue was purified by MPLC chromatography (silica gel, ethyl acetate/cyclohexane 0–100%, 49 min) to give 13 mg (15%) of product. ^1^H NMR (400 MHz, DMSO-*d*_6_): δ 12.1 (br. S, 1 H), 7.70 (d, *J* = 8.07 Hz, 2 H), 7.63 (s, 1 H), 7.44 (br. s., 1 H), 7.32 (t, *J* = 7.6 Hz, 2 H), 7.00–7.12 (m, 2 H), 6.92–6.98 (m, 3); MS (ESI): 237.1 [M + H]^+^; HPLC: t_R_ = 0.74 (94%).

#### Synthesis of compound 7

1-methyl-4-(4-phenoxyphenyl)-1H-imidazole. Analogous to the synthesis of compound **6** with 4-bromo-1-methyl-1H-imidazole (100 mg, 0.621 mmol) and (4-phenoxyphenyl)boronic acid (266 mg, 1.242 mmol). Yield: 155 mg (56%). ^1^H NMR (400 MHz, DMSO-*d*_6_): δ 7.67 (d,*J* = 8.7 Hz, 2 H), 7.54 (s, 1 H), 7.47 (s, 1 H), 7.29–7.36 (m, 2 H), 7.04–7.12 (m, 1 H), 6.94 (t, *J* = 8.4 Hz, 4 H), 3.61 (s, 3 H); MS (ESI): 251.1 [M + H]^+^; HPLC: t_R_ = 0.78 (100%).

#### Synthesis of compound 8

3-(4-phenoxyphenyl)-1H-pyrazol-5-ol. Ethyl 3-oxo-3-(4-phenoxyphenyl)propanoate (100 mg, 0.35 mmol) and hydrazine hydrate (0.019 ml, 0.39 mmol) were dissolved in 2 ml of acetic acid and heated to reflux (120 °C) for 3 h. The reaction mixture was cooled down to rt, a white solid precipitated. The solid was filtered off and washed with Et_2_O and dried under hv. The product was used without further purification. Yield: 70 mg (77%) of a white solid. ^1^H NMR (400 MHz, DMSO-*d*_6_): δ 12.01 (br. s., 1 H), 7.67 (d, *J* = 8.07 Hz, 2 H), 7.41 (t, *J* = 7.40 Hz, 2 H), 7.09–7.24 (m, 1 H), 7.04 (br. s., 4 H), 5.83 (br. s., 1 H); MS (ESI): 253.1 [M + H]^+^; HPLC: t_R_ = 0.88.

#### Synthesis of compound 9

**Step 1:** 33-(4-phenoxyphenyl)-1-(triisopropylsilyl)-1H-pyrrole. Analogous to the synthesis of compound **6** with 3-bromo-1-(triisopropylsilyl)-1H-pyrrole (200 mg, 0.662 mmol) and (4-phenoxyphenyl)boronic acid (283 mg, 1.323 mmol). Yield: 259 mg (27%). MS (ESI): 392.2 [M + H]^+^; HPLC: t_R_ = 1.74.

**Step 2:** TFA (98% in H_2_O, 2.5 ml, 32.5 mmol) was added dropwise over 5 min to 3-(4-phenoxyphenyl)-1H-pyrrole. 3-(4-phenoxyphenyl)-1-(triisopropylsilyl)-1H-pyrrole (173 mg, 0.442 mmol) and stirred for 1.5 h at rt. The rxn mixture was basified with saturated NaHCO_3_ solution, diluted with dichloromethane and washed with water. The aqueous layer was three times extracted with dichloromethane. The combined organics were dried over Na_2_SO_4_, filtered and concentrated. The reaction mixture was purified via MPLC chromatography (silica gel, ethyl acetate/cyclohexane 0–35%, 10 min) followed by SFC chromatography. ^1^H NMR (400 MHz, DMSO-*d*_6_): δ 10.83 (br. s., 1 H), 7.47 (d, *J* = 9 Hz, 2 H), 7.30 (t, *J* = 8 Hz, 2 H), 7.01–7.12 (m, 2 H), 6.92 (d, *J* = 8 Hz, 2 H), 6.89 (d, *J* = 9 Hz, 2 H), 6.72 (m, 1 H), 6.33 (m, 1 H); MS (ESI): 236.1 [M + H]^+^; HPLC: t_R_ = 1.18 (98%).

#### Synthesis of compound 16

**Step 1:** 2-((2-(diethoxymethyl)pyrimidin-5-yl)oxy)benzo[d]thiazole. 2-Chlorbenzothiazole (0.365 ml, 2.95 mmol) was dissolved in 13 ml of DMF and flushed with argon. To this solution 2-(diethoxymethyl)pyrimidin-5-ol (439 mg, 2.21 mmol) and Cs_2_CO_3_ (2881 mg, 8.84 mmol) were added. The reaction mixture (yellow suspension) was stirred at 80 °C for 1.5 h. LC/MS: the reaction was complete.

The rxn mixture was filtered and diluted with EtOAc (90 ml) and washed with NaHCO_3_-solution (45 ml) and brine (35 ml). The aqueous layers were extracted with EtOAc (2 × 60 ml). The combined organics were dried with Na_2_SO_4_, filtered off and evaporated. The crude product was purified by MPLC chromatographie (silica gel ethyl acetate/cyclohexane: 0–40%, 23 min). Yield: 680 mg (66%) of a yellow oil. ^1^H NMR (600 MHz, DMSO-*d*_6_): δ 9.12 (s, 2 H), 8.01 (d, *J* = 9 Hz, 1 H), 7.73 (d, *J* = 9 Hz, 1 H), 7.46 (t, *J* = 9 Hz, 1 H), 7.38 (t, *J* = 9 Hz, 1 H), 5.58 (s, 1 H), 3.70–3.77 (m, 2 H), 3.6–3.65 (m, 2 H), 1.17 (t, *J* = 7.5 Hz, 6 H); MS (ESI): 332.2 [M + H]^+^; HPLC: t_R_ = 1.11 (89%).

**Step 2:** 2-((2-(diethoxymethyl)pyrimidin-5-yl)oxy)benzo[d]thiazole (680 mg, 2.052 mmol) was dissolved in a mixture of dichloromethane, water and trifluoracetic acid (15/2.5/2.5 ml). The resulting colorless emulsion was stirred for 5 h at rt. Additional 2.5 ml of trifluoracetic acid and 5 m l of 2 M HCl was added and the reaction mixture was stirred for 5 h at 55 °C. Then the mixture was diluted with dichloromethane, washed with water and brine, dried over Na_2_SO_4_, filtered and evaporated. The crude product was purified by MPLC chromatography (silica gel, ethyl acetate/cyclohexane 0–50%, 28 min). Yield: 346 mg (54%). ^1^H NMR (600 MHz, DMSO-*d*_6_): δ 10.02 (s, 1 H), 9.38 (s, 2 H), 8.06 (d, *J* = 9 Hz, 1 H), 7.76 (d, *J* = 9 Hz, 1 H), 7.49 (t, *J* = 9 Hz, 1 H), 7.42 (t, *J* = 9 Hz, 1 H); MS (ESI): 258.1 [M + H]^+^, 276.1 [M + H2O]^+^; HPLC: t_R_ = 0.70.

#### Synthesis of compound 17

5-(thiazolo[4,5-b]pyridin-2-yloxy)picolinaldehyde. 2-Chlorothiazolo[4,5-b]pyridine (693 mg, 4.06 mmol) was dissolved in 10 ml of N,N-dimethylformamid and flushed with argon. 5-hydroxypicolinaldehyde (250 mg, 2.03 mmol) and Cs2CO3 (1.985 g, 6.09 mmol) were added and the mixture was stirred for 2 h at 80 °C. The reaction mixture was diluted with 150 ml of ethyl acetate and washed with saturated NaHCO_3_ solution, water and brine. The organic layers were dried over Na_2_SO_4_, filtrated and evaporated. The crude product was purified by MPLC (silica gel, ethyl acetate/heptane 10–100%, 45 min).Yield: 114 mg (22%) of yellow crystals. ^1^H NMR (400 MHz, DMSO-*d*_6_): δ 10.04 (s, 1 H) 9.05 (m, 1 H), 8.57 (d, *J* = 6 Hz, 1 H), 8.52 (d, *J* = 8 Hz, 1 H), 8.32 (m, 1 H), 8.15 (d, *J* = 8 Hz, 1 H), 7.41 (dd, *J* = 6 and 8 Hz, 1 H); MS (ESI): 258.0 [M + H]^+^; HPLC: t_R_ = 0.72 (98%).

## Electronic supplementary material

Supplementary Information
