# Supplementary material for: Feasibility and physiological relevance of designing highly potent aminopeptidase-sparing leukotriene A4 hydrolase inhibitors
Source: Sci Rep. 2017 Oct 19;7:13591. doi: 10.1038/s41598-017-13490-1 (PMC5648829; doi:10.1038/s41598-017-13490-1)
Supplement: Supplementary file 1 — Supplementary Information [file 41598_2017_13490_MOESM1_ESM.pdf]

Supplementary Information

Feasibility and physiological relevance of designing highly potent aminopeptidase-sparing leukotriene A4 hydrolase inhibitors

Shin Numao[1], Franziska Hasler[2], Claire Laguerre[3], Honnappa Srinivas[1], Nathalie Wack[2], Petra Jäger[2], Andres Schmid[1], Arnaud Osmont[3], Patrik Röthlisberger[1], Jeremy Houguenade[2], Christian Bergsdorf[1], Janet Dawson[2], Nathalie Carte[3], Andreas Hofmann[3], Christian Markert[4], Liz Hardaker[5], Andreas Billich[2], Romain M. Wolf[4], Carlos A. Penno[3], Birgit Bollbuck[4], Wolfgang Miltz[4], Till A. Röhn[2]*

[1]Chemical Biology & Therapeutics,[2]Autoimmunity, Inflammation and Transplantation, [3]Analytical Sciences & Imaging, [4]Global Discovery Chemistry, and [5]Respiratory Research, Novartis Institutes for BioMedical Research, Novartis Pharma AG, Basel, Switzerland.

*Corresponding author

Supplementary Methods

LTA4H substrates used in enzymatic assays

While the reported natural substrates for LTA4H are LTA4 and PGP, in literature, several synthetic substrates have been used as surrogates due the difficulty in using LTA4 or PGP in high throughput assays. The most frequently used substrate is alanine coupled to a fluorophore (example, Ala-AMC), which is used as a surrogate substrate of PGP hydrolysis. This substrate allows for reaction progression to be measured by an increase in fluorescence signal. Interestingly, it has been reported that certain compounds activate Ala-AMC hydrolysis, and this has been used as an indication that the PGP hydrolysis activity[12,13] is enhanced. Additional structural studies of LTA4H bound to peptide substrates suggested that an arginine could bind to the S1 pocket of LTA4H[17]. We therefore reasoned that a substrate whereby a fluorescence group is coupled to arginine (example, Arg-Rho110-D-Pro (Arg-Rho) and Arg-AMC) would also act as substrates for LTA4H. Furthermore, we reasoned that because arginine

sidechain would extend deep into the LTA4 binding pocket, hydrolase inhibitors would also inhibit an assay using either the Arg-Rho110-D-Pro (Arg-Rho) or the Arg-AMC as substrates.

Crystal data collection and refinement

Supplementary Table 1. Crystal data collection and refinement statistics

	LTA4H E296Q-PGP	LTA4H-Compound 11	LTA4H-compound 15
A. *Data collection*			
Space group	$P2_12_12_1$	$P2_12_12_1$	$P2_12_12_1$
Unit-cell dimensions (Å)	*a* = 77.4 *b* = 87.5 *c* = 99.0	*a* = 78.0 *b* = 87.1 *c* = 99.1	*a* = 78.4 *b* = 87.1 *c* = 99.5
Resolution (Å)	50–1.84	50–1.95	50–2.24
Observations; unique reflections	387899, 59014	274809, 49717	181827, 33323
Completeness(%)	99.8 (99.9)*	99.8 (99.9)*	99.8 (99.9)*
I/σ	13.02 (2.30)*	13.02 (2.30)*	13.02 (2.30)*
R_{merge}	11.6 (73.6)*	11.6 (73.6)*	11.6 (73.6)*
B. *Refinement statistics*			
Resolution (Å)	29–1.84	24–1.95	24–2.24
Reflections	59014	49716	33322
R-factor, R_{free}	15.6, 18.6	16.2, 19.4	16.5, 21.6
Total number of protein atoms	4871	4860	4852
Water atoms, and other heteroatoms	676, 688	689, 732	561, 596
RMS bonds (Å), angles (deg.)	0.01, 1.00°	0.01, 1.00°	0.01, 1.10°

*Numbers in parentheses refer to the highest resolution shell

Molecular dynamics settings:

MD starting coordinates for protein/ligand complexes were generated from the PDB entries 4MS6 (PGP analogue) and internal x-ray structures (for PGP, partial occupancy in E296Q mutated LTA4H data). Protein and peptide parts were assigned Amber force field parameters of the ff14SB parameter set[47]. Organic ligands like e.g. cpd. 17 were treated with the GAFF force field[48], with partial charges computed via AM1/BCC[49].

The tetrahedral coordination of Zn^{2+} ion was treated with the CaDA approach[50,]. To conserve the tetrahedral arrangement around the zinc, both H295 and H299 had to be converted to 'histidinate' (i.e.,

negatively charged) to keep a constant distance to the respective pseudo atoms on Zn^{2+}. The excess negative charge thus introduced was compensated by protonating E271 and E318. Since the standard MD by force fields cannot handle reactions anyway, this situation may be considered as a momentary occurrence and should not affect the conclusions drawn from the short MD simulations here. The starting arrangement for MD is shown in supplementary figure 1.

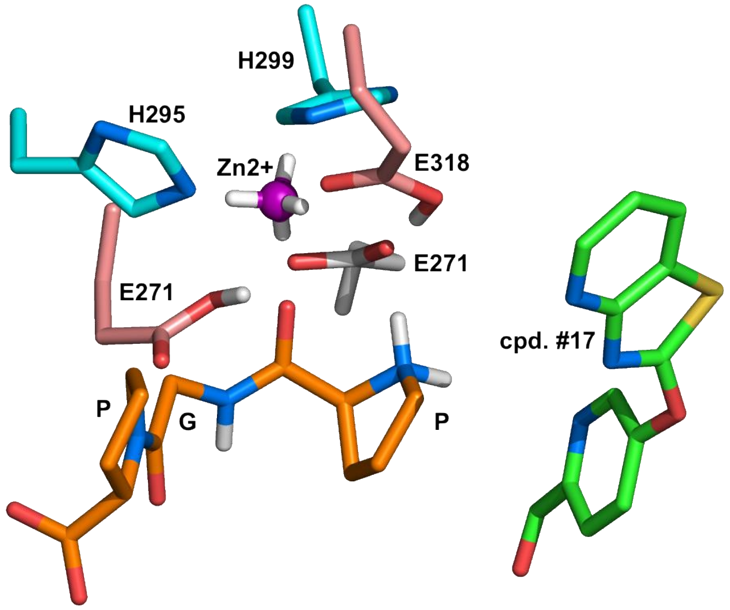

Supplementary Figure 1 . Settings around the zinc active site used for molecular dynamics: PGP in orange, compound 17 in green, protonated glutamic acids E271 and E318 in pink, histidinates H295 and H299 in light blue, zinc cation in magenta with the four pseudo atoms bearing each one fourth of the total +2 charge in white.

MD runs (protein alone, ligand alone, and complex) were executed with explicit water (TIP3 water, periodic boundary conditions). The overall charge was neutralized by addition of sodium cations. Simulations were carried out by the sander. MPI module of AmberTools, using 8 processors in parallel. The non-bonded cutoff distance was set to 8 Å. The entire system was minimized for 1000 steps conjugated gradient, followed by a heat-up to 300 K over 100 picoseconds (ps) at constant volume (NVT), then equilibrated for 100 ps at constant pressure (NPT) to adjust the density. Finally, the production trajectory was run at constant volume (NVT) for 5 ns (using integration steps of 2 femtoseconds with SHAKE switched on). The temperature was regulated via Langevin dynamics to 300 K.

Supplementary results

Trajectories were analyzed with the *cpptraj* module of AmberTools[51]. In supplementary figure 2 (left) are shown as overly 50 equidistant frames (spaced by 100 ps) from the MD trajectory of the LTA4H/PGP/compound 17 ternary complex. The motion of the compound 17 is more important than that of PGP which follows obviously from the less defined interactions with LTA4H. In supplementary figure 2(right) are depicted the same 50 frames (PGP as solid molecular surface, compound 17 as gridded surface). Obviously, both structures share a common portion of the binding site, although of course during the ternary complex MD, van der Waals repulsion avoids momentary overlap.

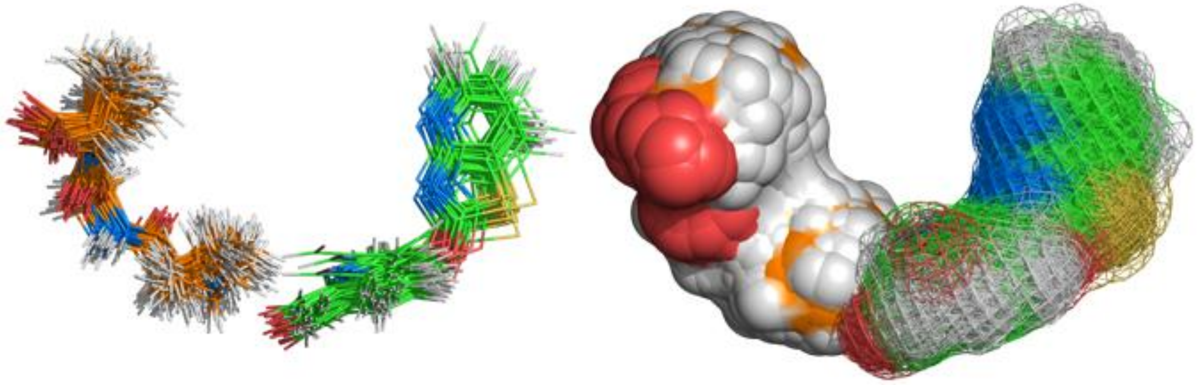

Supplementary Figure 2. on the left, overlay of 50 equidistant (by 100 ps) MD frames of PGP and compound 17 form the MD trajectory of the ternary complex LTA4H/PGP/cpd.17; on the right, surface representation of the 50 overlaid frames, indicating the (overall) common occupancy of a part of the binding site (see also text).

The effect of “too close for comfort” is best rendered by comparing the per-residue atomic fluctuations computed from the ternary complex and for individual LTA4H/PGP and LTA4H/cpd.17 MD trajectories (Supplementary Table 2). In the ternary complex, the fluctuations for both PGP and compound 17 are smaller than for the individual complexes with PGP or cpd. 17 alone. Only the C-terminal proline in PGP is not affected by the presence or absence of compound 17. These results are also shown graphically in Supplementary Figure 3, after converting the atomic fluctuations to B-factors.

**Supplementary Table 2**: Atomic fluctuations (Å) "per-residue" for the heavy atoms in PGP and/or cpd.17 during the 5 ns MD trajectories of the respective complexes.				
complex	PRO (N-terminal)	GLY	PRO (C-terminal)	cpd. 17
LTA4D/PGP/cpd.17	0.458	0.484	0.574	0.656
LTA4H/PGP	0.649	0.547	0.579	
LTA4H/cpd.17				0.848

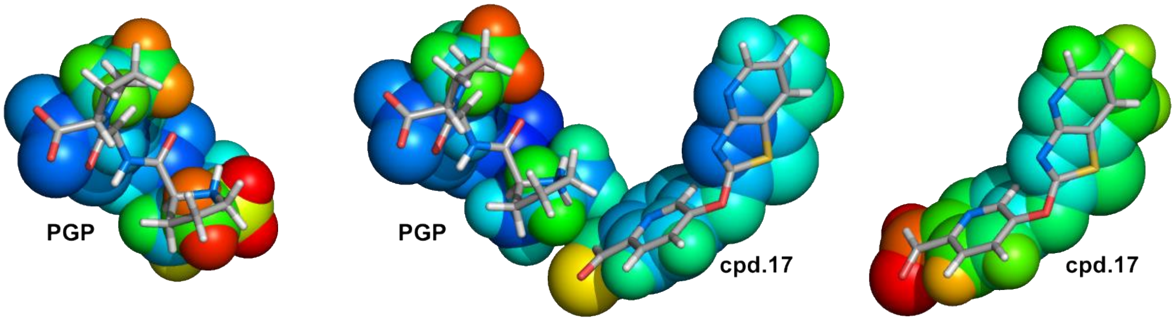

Supplementary Figure 3. B-factor colouring of PGP and cpd.17 (PyMOL [9], using "spectrum b, minimum=0, maximum=50", with the default colouring from blue/cold to red/hot); B-factor values were computed from MD trajectories using cpptraj [8]; left: for the LTA4/PGP complex; middle: for ternary complex LTA4H/PGP/cpd.17; right: for the LTA4H/cpd.17 complex.

Both Supplementary Table 2 and Supplentary Figure 3 clearly show that the ternary complex induces motional constraints on PGP and the additional ligand in the extended hydrolase pocket. It can therefore be assumed that an additional ligand like compound 17 in this example also affects (partially inhibits) the hydrolysis of PGP.

Bibliography

12. Jiang X, Zhou L, Wei D, Meng H, Liu Y, Lai L. Activation and inhibition of leukotriene A4 hydrolase aminopeptidase activity by diphenyl ether and derivatives. *Bioorg Med Chem Lett* **18**, 6549-6552 (2008).

13. De Oliveira EO*, et al.* Effect of the leukotriene A4 hydrolase aminopeptidase augmentor 4-methoxydiphenylmethane in a pre-clinical model of pulmonary emphysema. *Bioorg Med Chem Lett* **21**, 6746-6750 (2011).

17. Tholander F, Muroya A, Roques BP, Fournie-Zaluski MC, Thunnissen MM, Haeggstrom JZ. Structure-based dissection of the active site chemistry of leukotriene A4 hydrolase: implications for M1 aminopeptidases and inhibitor design. *Chem Biol* **15**, 920-929 (2008).

47. Maier JA, Martinez C, Kasavajhala K, Wickstrom L, Hauser KE, Simmerling C. ff14SB: Improving the Accuracy of Protein Side Chain and Backbone Parameters from ff99SB. *J Chem Theory Comput* **11**, 3696-3713 (2015).

48. Wang J, Wolf RM, Caldwell JW, Kollman PA, Case DA. Development and testing of a general amber force field. *J Comput Chem* **25**, 1157-1174 (2004).

49. Jakalian A, Jack DB, Bayly CI. Fast, efficient generation of high-quality atomic charges. AM1-BCC model: II. Parameterization and validation. *J Comput Chem* **23**, 1623-1641 (2002).

50. Pang Y-P. Novel zinc protein molecular dynamics simulations: Steps toward anti-angiogenesis for cancer treatment. *J Mol Model* **5**, 196-202 (1999).

51. Roe DR, Cheatham TE, 3rd. PTRAJ and CPPTRAJ: Software for Processing and Analysis of Molecular Dynamics Trajectory Data. *J Chem Theory Comput* **9**, 3084-3095 (2013).
